# Statin use is associated with a lower risk of recurrence after curative resection in BCLC stage 0-A hepatocellular carcinoma

**DOI:** 10.1186/s12885-021-07796-7

**Published:** 2021-01-15

**Authors:** Shih-Yu Yang, Chih-Chi Wang, Kuang-Den Chen, Yueh-Wei Liu, Chih-Che Lin, Ching-Hui Chuang, Yu-Chieh Tsai, Chih-Chien Yao, Yi-Hao Yen, Chang-Chun Hsiao, Tsung-Hui Hu, Ming-Chao Tsai

**Affiliations:** 1 Division of Hepato-Gastroenterology, Department of Internal Medicine, Kaohsiung Chang Gung Memorial Hospital and Chang Gung University College of Medicine, 123 Ta Pei Road, Kaohsiung, Taiwan; 2 Liver Transplantation Center and Department of Surgery, Kaohsiung Chang Gung Memorial Hospital and Chang Gung University College of Medicine, Kaohsiung, Taiwan; 3 Center for Translational Research in Biomedical Sciences, Liver Transplantation Program and Department of Surgery, Kaohsiung Chang Gung Memorial Hospital and Chang Gung University College of Medicine, Kaohsiung, Taiwan; 4 Head Nurse, Department of Nursing, Kaohsiung Chang Gung Memorial Hospital, Kaohsiung, Taiwan; 5 Graduate Institute of Clinical Medical Sciences, College of Medicine, Chang Gung University, Taoyuan, Taiwan; 6 Division of Pulmonary and Critical Care Medicine, Department of Medicine, Kaohsiung Chang Gung Memorial Hospital and Chang Gung University College of Medicine, Kaohsiung, Taiwan

**Keywords:** Hepatocellular carcinoma, Statin, Resection, Recurrence

## Abstract

**Background:**

Use of statins is associated with a reduced risk of hepatocellular carcinoma (HCC). However, the effect of statin use on HCC recurrence is unclear. This study aimed to evaluate the effect of statin use on recurrence after curative resection among patients with HCC.

**Methods:**

We retrospectively assessed 820 patients with Barcelona Clinic Liver Cancer (BCLC) stage 0 or A HCC who underwent primary resection between January 2001 and June 2016 at Kaohsiung Chang Gung Memorial Hospital. Exposure to statins was defined as use of a statin for at least 3 months before HCC recurrence. Factors that influenced overall survival (OS) and recurrence-free survival (RFS) were analyzed using Cox proportional hazards models.

**Results:**

Of the 820 patients, 46 (5.6%) used statins (statin group) and 774 (94.4%) did not (non-statin group). During the mean follow-up of 76.5 months, 440 (53.7%) patients experienced recurrence and 146 (17.8%) patients died. The cumulative incidence of HCC recurrence was significantly lower in the statin group than the non-statin group (*p* = 0.001); OS was not significantly different between groups. In multivariate analysis, age (hazard ratio [HR]: 1.291; *p* = 0.010), liver cirrhosis (HR: 1.743; *p* < 0.001), diabetes (HR:1.418; *p* = 0.001), number of tumors (HR: 1.750; *p* < 0.001), tumor size (HR: 1.406; *p* = 0.004) and vascular invasion (HR: 1.659; *p* < 0.001) were independent risk factors for HCC recurrence, whereas statin use (HR: 0.354; *p* < 0.001) and antiviral therapy (HR: 0.613; *p* < 0.001) significantly reduced the risk of HCC recurrence. The statin group still had lower RFS than the non-statin group after one-to-four propensity score matching.

**Conclusion:**

Statins may exert a chemo-preventive effect on HCC recurrence after curative resection.

**Supplementary Information:**

The online version contains supplementary material available at 10.1186/s12885-021-07796-7.

## Background

Hepatocellular carcinoma (HCC), the most common primary malignancy of the liver, is the second leading cause of cancer-related deaths in many regions of the world [1]. Approximately 850,000 new cases of HCC are diagnosed worldwide per year [2]. The main risk factors for HCC are chronic infection with hepatitis B virus (HBV) or hepatitis C virus (HCV), consumption of aflatoxin-contaminated foodstuffs, heavy alcohol intake, obesity, smoking and type 2 diabetes [3]. The current management strategies for HCC depend on the tumor stage and include surgical resection, liver transplantation, radiofrequency ablation (RFA), transarterial chemoembolization, radiation therapy and systemic therapy [4, 5]. The ideal candidates for resection are patients with early stage (BCLC stage 0 or A) who do not have extrahepatic metastasis, macrovascular invasion or clinically significant portal hypertension [6].

Surgical resection is a potentially curative treatment for HCC, though cumulative recurrence rates remain high (50–60%) [7–10]. Known risk factors for HCC recurrence after hepatectomy are tumor size, serum α-fetoprotein, tumor differentiation, microvascular invasion, cirrhosis, surgical margin, serum HBV viral load and metabolic syndrome [7, 9, 11–13]. Nucleos(t)ide analogue (NA) therapy may reduce the risk of HCC recurrence after hepatic resection among patients with HBV-related HCC [14]. The adjuvant therapy sorafenib, a targeted therapy for advanced HCC, has been proven not to prevent HCC recurrence after complete resection or ablation of primary HCC [15]. However, using NA therapy alone is not enough to prevent HCC recurrence. To decrease the risk of HCC recurrence after curative resection, other effective chemopreventive agents need to be identified.

Statins, cholesterol-lowering 3-hydroxy-3-methyglutaryl-coenzyme A (HMG-CoA) reductase inhibitors, are the most common medications used for primary and secondary prevention of cardiovascular disease and mortality [16]. In addition to their effect on cholesterol biosynthesis, numerous previous studies have indicated statins can exert chemopreventive effects and reduce the risk of HCC in individuals with HBV [17, 18] or HCV [19, 20] infection. In vitro studies and animal models have explored the mechanisms underlying the anticancer effects of statins in HCC [21, 22]. Although these studies demonstrate statins reduce the risk of developing HCC, few studies have explored the impact of statins on the outcome of patients with HCC after curative resection. Thus, we aimed to evaluate the effect of statin use on the risk of recurrence after curative resection in patients with HCC.

## Methods

### Study design

**Fig. 1:**
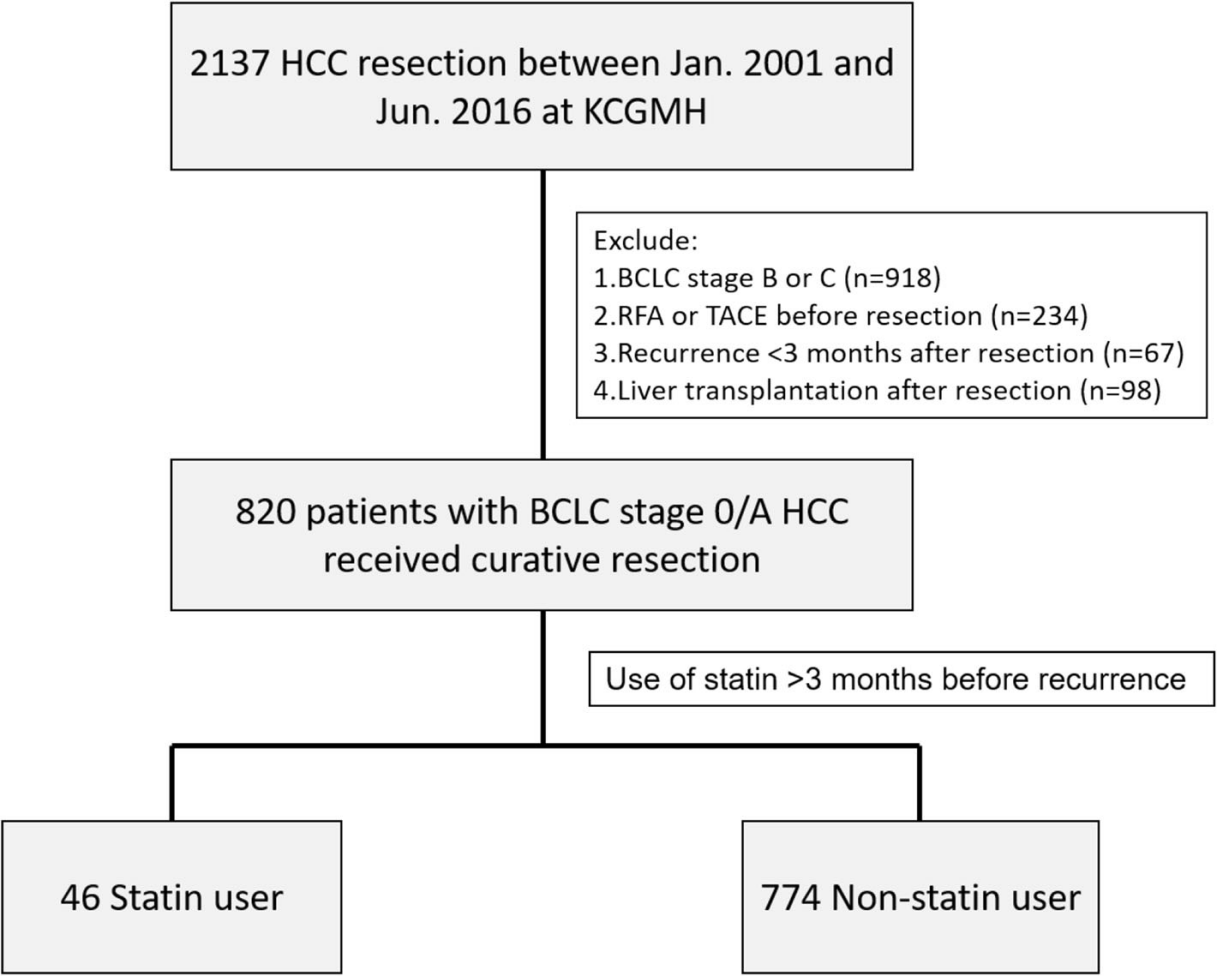
Patient selection flow diagram

The data used in this study were extracted from the Kaohsiung Chang Gung Memorial Hospital HCC registry database. A total of 2137 patients diagnosed with HCC who underwent surgical resection between January 2001 and June 2016 at Kaohsiung Chang Gung Memorial Hospital were retrospectively enrolled. We excluded 918 patients with Barcelona Clinic Liver Cancer (BCLC) stage B or C, 234 patients who underwent prior treatment for HCC and 67 patients who developed recurrence within less than 3 months after resection. In well-selected patients, liver transplantation is generally considered to cure the tumor and underlying cirrhosis at the same time, thus strongly influences survival and recurrence [23]. Therefore, 98 patients who underwent salvage liver transplantation were also excluded. Finally, a total of 820 patients with BCLC stage 0 or A HCC who underwent primary curative resection (Fig. 1) were included in this study.

This study was conducted in accordance with the standards of the Declaration of Helsinki and current ethics guidelines; approval was obtained from the Ethics Committee of Chang Gung Memorial Hospital (IRB number: 201901103B0). The requirement for informed consent was waived by the IRB; all data were analyzed anonymously.

### Exposure to chemopreventive agents

To define the statins group and non-statins group, we calculated the defined daily dose (DDD) recommended by the World Health Organization to measure the amount of drugs prescribed [24]. Cumulative DDD (cDDD) was estimated as the sum of the dispensed DDDs for any statin (namely Atorvastatin, Fluvastatin, Pitavastatin and Rosuvastatin) before HCC recurrence. Patients taking a statin cDDD of more than 90 were enrolled in the statins group; patients with statin cDDD of less than 90 were enrolled in the non-statins group. The cDDD for other chemopreventive agents including aspirin, NSAIDs (namely diclofenac, ibuprofen, indomethacin, mefenamic acid, aceclofenac, sulindac, celecoxib, etoricoxib and naproxen), and metformin were also recorded.

### Study assessments and follow-up evaluation

Medical records were reviewed to obtain data on patient demographics and clinical characteristics, including serum biochemistry, albumin, alpha-fetoprotein (AFP), Child-Pugh classification, viral hepatitis status, duration of follow-up and outcomes. The diagnosis of cirrhosis was confirmed using the histopathology reports for surgically resected non-tumor tissues. HCC stage was defined according to the BCLC guidelines [25]. Tumor differentiation was assessed using the Edmondson grading system.

Patients were followed-up 1 month after surgery, every 3 months in the first year, and every 3–6 months in subsequent years. Serum AFP levels, serum biochemistry and abdominal ultrasonography were performed at every follow-up. Dynamic computed tomography or magnetic resonance studies were performed 1 month after resection and every 12 months thereafter, or if HCC recurrence was clinically suspected. Last follow-up was April 30, 2020. Recurrence-free survival (RFS) was defined as the interval between surgery and the date of diagnosis of the first recurrence; overall survival (OS), as the interval between surgery and death or last follow-up.

### Statistical analysis

Propensity score matching (PSM) was applied to reduce selection bias between the study groups. Age, sex, diabetes mellitus, hepatitis B, hepatitis C, liver cirrhosis, Child-Pugh grade, tumor size, tumor number and microvascular invasion were selected as independent variables. The greedy method of NCSS 10 Statistical Software (LLC, Kaysville, UT, USA) was used for matching the study groups in a 1:4 ratio; the caliper width was 0.2 of the standard deviation of the propensity score between study groups. The standardized mean difference (SMD) was used to evaluate covariate balance after PSM.

Demographic data were compared between groups using Fisher’s exact test or the chi-square test, as appropriate. Continuous variables are expressed as the median ± interquartile range (IQR). The Kaplan-Meier method was used to plot the RFS and OS curves stratified by chemopreventive agent use and the curves were compared using the log-rank test. Factors that were significant in the univariate analysis (*p* < 0.05) were included in multivariate analyses of OS and RFS using a Cox forward stepwise variable selection process. Hazard ratios (HR) and 95% confidence intervals (CI) were also calculated for each factor. Statistical analyses were performed using SPSS 22.0 software (SPSS Inc., Chicago, IL, USA). All statistical tests were two-sided; *p*-values < 0.05 were considered significant.

## Results

### Comparison of the clinical characteristics of patients with and without statin use

Table 1 summarizes the characteristics of the study cohort, which included 639 males and 181 females, with an age range of 52–66-years-old and median age of 59. Overall, 222 patients (27.1%) had diabetes before surgery and 378 (46.1%) were diagnosed with cirrhosis. Cirrhosis was defined as METAVIR stage 4 fibrosis based on histopathological evaluation of resected non-tumor liver tissues [26].

**Table 1:** Comparison of clinical and pathological characteristics before hepatectomy for patients with or without statin use

	Total (*n* = 820)	Statin (*n* = 46)	Non-statin (*n* = 774)	*P*-value
Age (years; median, IQR)	58.8 (52–66)	62 (58–63)	58.6 (52–66)	0.037
Age (> 60 years), *n* (%)	432 (52.7%)	31 (67.4%)	401 (51.8%)	0.04
Male, *n* (%)	639 (77.9%)	40 (87.0%)	599 (77.4%)	0.129
Bilirubin (g/dL; median, IQR)	0.8 (0.6–1.0)	0.8 (0.5–1.0)	0.8 (0.6–1.0)	0.556
Albumin (g/dL; median, IQR)	3.7 (3.2–4.1)	3.8 (3.5–4.2)	3.6 (3.2–4.1)	0.129
AFP (> 200 ng/mL), *n* (%)	145 (18.2%)	6 (14.6%)	139 (18.4%)	0.547
Liver cirrhosis, *n* (%)	378 (46.1%)	12 (26.1%)	366 (47.3%)	0.005
Hepatitis B, *n* (%)	458 (55.9%)	22 (47.8%)	436 (56.3%)	0.259
Hepatitis C, *n* (%)	284 (34.6%)	14 (30.4%)	270 (34.9%)	0.538
Diabetes, *n* (%)	214 (26.1%)	27 (58.7%)	187 (24.2%)	< 0.001
Tumor size (> 2 cm), *n* (%)	614 (74.9%)	42 (91.3%)	572 (73.9%)	0.008
Tumor number (single:multiple)	750:70	44:2	706:68	0.296
Child-Pugh grade (A:B)	752:68	42:4	710:64	0.919
Microvascular invasion, *n* (%)	302 (36.8%)	22 (47.8%)	280 (36.2%)	0.112
Histological grade (well:moderate:poor)	107:684:19	6:38:1	101:646:18	0.965
Recurrence, *n* (%)	440 (53.7%)	15 (32.6%)	425 (54.9%)	0.003
Death, *n* (%)	146 (17.8%)	7 (15.2%)	139 (18.0%)	0.637

*AFP* α-fetoprotein

Of the 820 patients, 46 (5.6%) were taking statins (statin group) and 774 (94.4%) were not taking statins (non-statin group). Compared to the non-statin group, the patients in the statin group were significantly older (*p* = 0.037) and had a higher frequency of diabetes mellitus (DM; *p* < 0.001) and larger tumors (*p* = 0.008), but a lower frequency of cirrhosis (*p* = 0.005). Overall, the statin group had a lower rate of recurrence (*p* = 0.003), though overall survival was not significant different between the statin and non-statin groups (*p* = 0.667).

### Factors associated with HCC recurrence

**Fig. 2:**
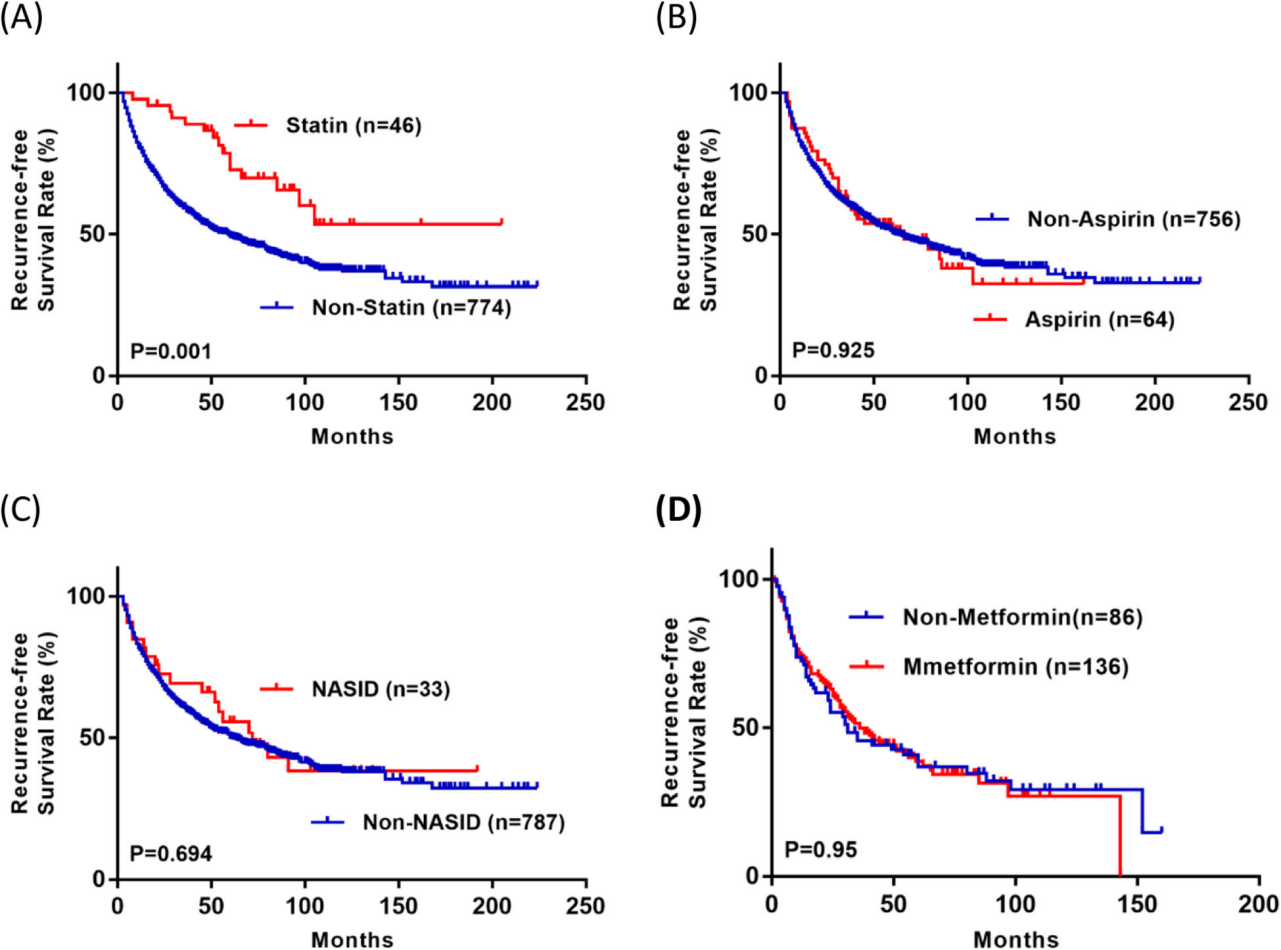
Kaplan-Meier cumulative recurrence-free survival curves for patients stratified by **a** statin use, **b** aspirin use, **c** NSAID use and **d** metformin use

**Fig. 3:**
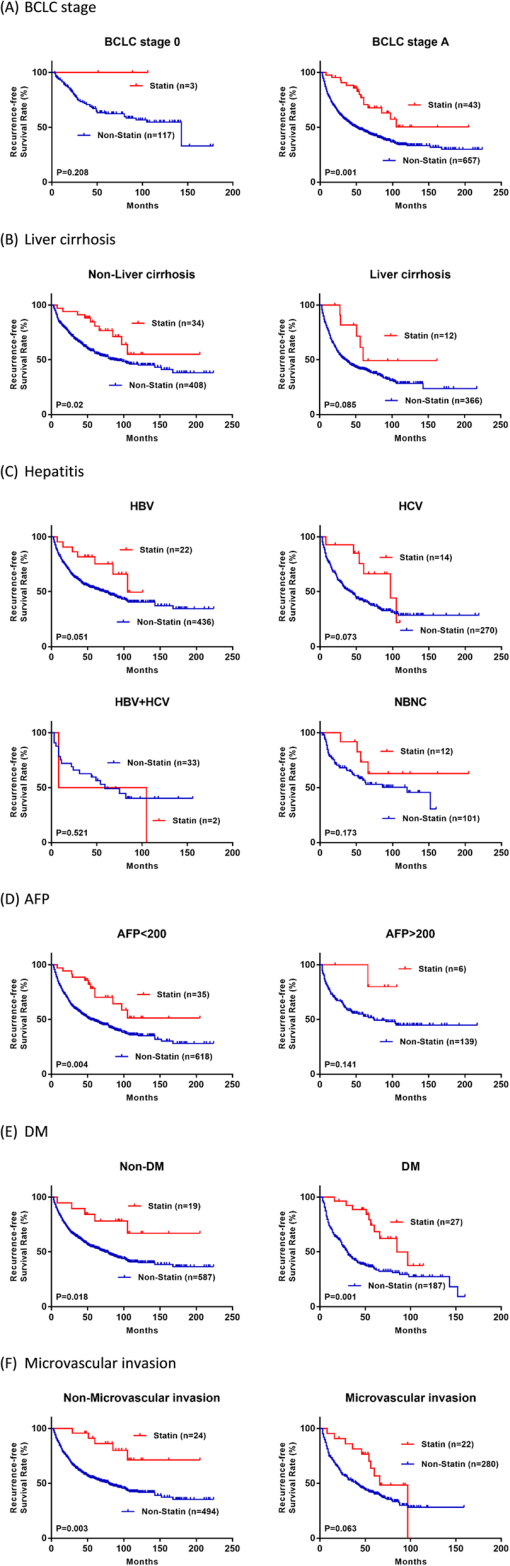
Kaplan-Meier cumulative recurrence-free survival curves for patients with or without statin use stratified by **a** BCLC stage, **b** liver cirrhosis, **c** hepatitis, **d** serum AFP, **e** diabetes mellitus and **f** microvascular invasion

A total of 440 (53.7%) patients developed recurrence during the mean follow-up period of 76.5 months. The Kaplan-Meier curves shown in Fig. 2 indicated statin use (*p* = 0.001) was associated with a significantly lower risk of HCC recurrence. In contrast, aspirin, NSAIDs and metformin were not significantly associated with HCC recurrence. In subgroup analysis based on various clinical characteristics (Fig. 3), RFS was significantly higher in the statin group than non-statin group in the subgroups of patients with BCLC stage A (*p* = 0.001), AFP < 200 ng/mL (*p* = 0.004), without cirrhosis (*p* = 0.02), CHB (*p* = 0.051), without DM (*p* = 0.018) and with DM (*p* = 0.001).

In the stepwise Cox proportional hazard model (Table 2), age (HR:1.291; CI: 1.064–1.566; *p* = 0.010), liver cirrhosis (HR: 1.743; CI: 1.437–2.113; *p* < 0.001), diabetes (HR:1.418; CI: 1.147–1.755; *p* = 0.001), multiple tumors (HR: 1.750; CI: 1.304–2.348; *p* < 0.001), tumor size > 2 cm (HR: 1.406; CI: 1.113–1.774; *p* = 0.004) and vascular invasion (HR: 1.659; CI: 1.364–2.018; *p* < 0.001) were independent risk factors for HCC recurrence. Moreover, statin use (HR: 0.354; CI: 0.210–0.599; *p* < 0.001) and antiviral therapy (HR: 0.613; CI: 0.503–0.748; *p* < 0.001) were associated with a significantly lower risk of HCC recurrence.

**Table 2:** Multivariate analysis of recurrence after curative hepatectomy for patients with BCLC 0/A stage HCC

Variable	Comparison	All (*n* = 820)		CHB (*n* = 458)		CHC (*n* = 284)	
		HR (95% CI)	*P*-value	HR (95% CI)	*P*-value	HR (95% CI)	*P*-value
Age (years)	>60 vs. ≦ 60	1.291 (1.064–1.566)	0.010				
Sex	Male vs. Female						
AFP (ng/mL)	>200 vs. ≦ 200						
Liver cirrhosis	Yes vs. No	1.743 (1.437–2.113)	< 0.001	1.991 (1.530–2.592)	< 0.001	1.617 (1.187–2.203)	0.002
Diabetes	Yes vs. No	1.418 (1.147–1.755)	0.001	1.823 (1.343–2.475)	< 0.001		
Child-Pugh grade	B vs. A						
Tumor number	Multiple vs. Single	1.750 (1.304–2.348)	< 0.001	1.582 (1.085–2.305)	0.017	2.091 (1.292–3.385)	0.003
Tumor size (cm)	>2 vs. ≦ 2	1.406 (1.113–1.774)	0.004	1.716 (1.221–2.411)	0.002		
Histology stages	Poor vs. well + moderate						
Vascular invasion	Yes vs. No	1.659 (1.364–2.018)	< 0.001	1.464 (1.120–1.913)	0.005	1.776 (1.309–2.409)	< 0.001
Statin	Yes vs. No	0.354 (0.210–0.599)	< 0.001	0.393 (0.181–0.854)	0.018		
NSAIDs	Yes vs. No						
Aspirin	Yes vs. No						
Metformin	Yes vs. No						
Antiviral therapy	Yes vs. No	0.613 (0.503–0.748)	< 0.001				
NA therapy	Yes vs. No			0.590 (0.453–0.768)	< 0.001		
HCV therapy	Yes vs. No					0.496 (0.361–0.682)	< 0.001

*NSAIDs* Nonsteroidal anti-inflammatory drugs, *NA therapy* Nucleos(t)ide analogues

HCV therapy included interferon and direct-acting antiviral medications

We further analyzed RFS in subgroup among CHB and CHC patients. Among CHB patients (*n* = 458), liver cirrhosis, diabetes, tumor number, tumor size and vascular invasion were independent risk factors for HCC recurrence. Statin and nucleos(t)ide analogues (NA) therapy were found to decrease HCC recurrence. Among CHC patients (*n* = 284), liver cirrhosis, tumor number and vascular invasion were significantly associated with HCC recurrence. HCV therapy was associated with a significantly lower risk of recurrence.

### Factors associated with overall survival

**Fig. 4:**
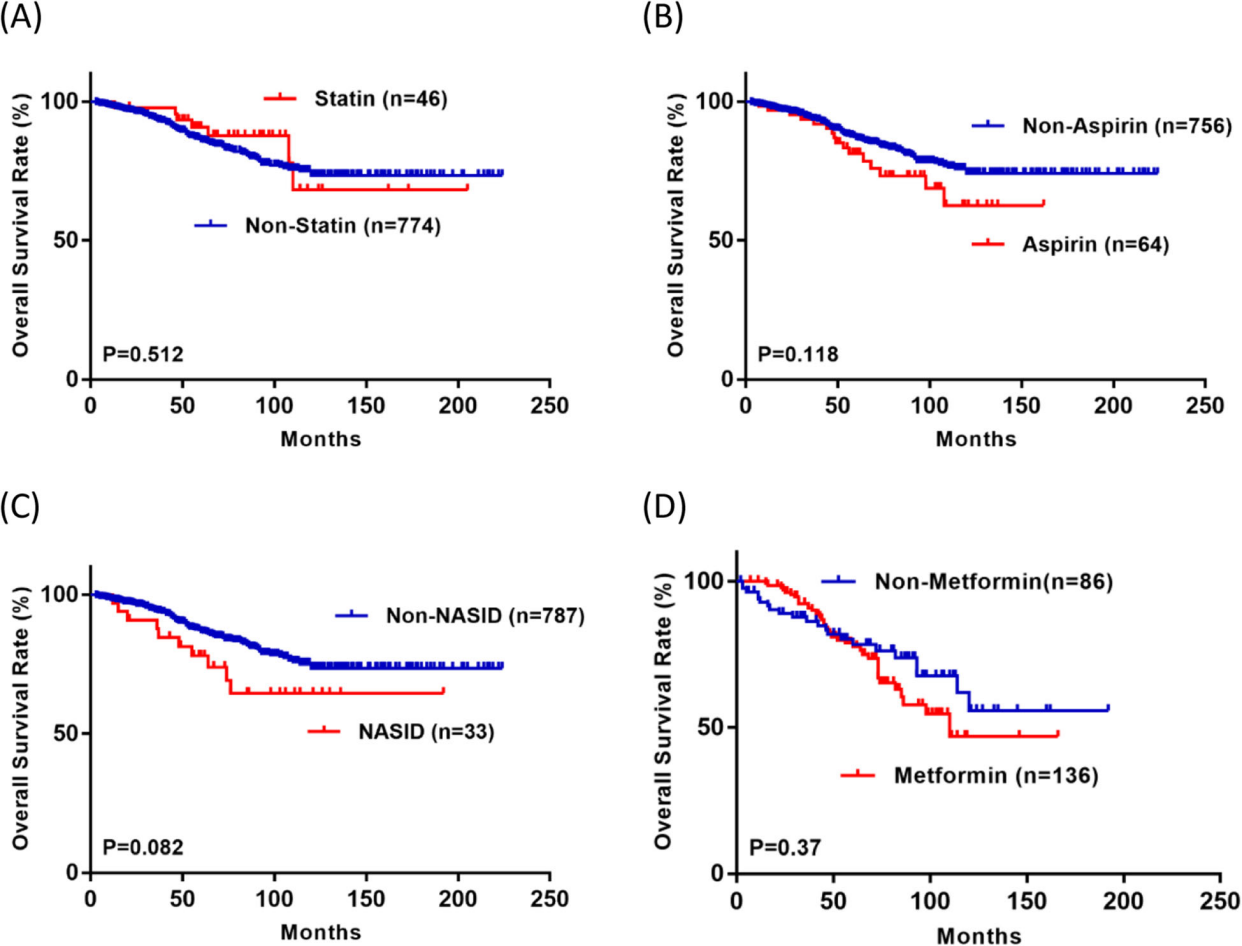
Kaplan-Meier cumulative overall survival curves for patients stratified by **a** statin use, **b** aspirin use, **c** NSAID use and **d** metformin use

A total of 146 (17.8%) patients died during follow-up. Overall, 91 (62.3%) patients died of liver-related causes: 82 of HCC and nine of complications associated with cirrhosis. Of the 55 patients who died of non-liver-related causes, 36 died of sepsis, 10 of malignancies other than HCC, three of out-of-hospital cardiac arrest, three of heart failure, one of intracranial hemorrhage, one of in-hospital cardiac arrest and one of acute respiratory distress syndrome. The Kaplan-Meier curves in Fig. 4 suggested that statin, aspirin, NSAID and metformin use were not associated with OS.

In the stepwise Cox proportional hazard model (Table 3), liver cirrhosis (HR: 1.644; CI: 1.180–2.290; *p* = 0.003), diabetes (HR: 2.064; CI: 1.478–2.881; *p* < 0.001), Child-Pugh grade (HR: 1.915; CI: 1.185–3.096; *p* = 0.008) and vascular invasion (HR: 2.339; CI: 1.670–3.276; *p* < 0.001) were related to poorer OS, whereas antiviral therapy (HR: 0.350; CI: 0.241–0.509; p < 0.001) were associated with better OS.

**Table 3:** Multivariate analysis of overall survival after curative hepatectomy for patients with BCLC 0/A stage HCC

Variable	Comparison	All (*n* = 820)		CHB (*n* = 458)		CHC (*n* = 284)	
		HR (95% CI)	*P*-value	HR (95% CI)	*P*-value	HR (95% CI)	*P*-value
Age (years)	>60 vs. ≦ 60						
Sex	Male vs. Female						
AFP (ng/mL)	>200 vs. ≦ 200						
Liver cirrhosis	Yes vs. No	1.644 (1.180–2.290)	0.003			1.828 (1.085–3.079)	0.023
Diabetes	Yes vs. No	2.064 (1.478–2.881)	< 0.001	2.633 (1.611–4.303)	< 0.001	1.940 (1.166–3.227)	0.011
Child-Pugh grade	B vs. A	1.915 (1.185–3.096)	0.008	2.223 (1.051–4.702)	0.037		
Tumor number	Multiple vs. Single						
Tumor size (cm)	>2 vs. ≦ 2						
Histology stages	Poor vs. well + moderate						
Vascular invasion	Yes vs. No	2.339 (1.670–3.276)	< 0.001	2.283 (1.403–3.716)	0.001	3.068 (1.808–5.206)	< 0.001
Statin	Yes vs. No						
NSAIDs	Yes vs. No						
Aspirin	Yes vs. No						
Metformin	Yes vs. No						
Antiviral therapy	Yes vs. No	0.350 (0.241–0.509)	< 0.001				
NA therapy	Yes vs. No			0.452 (0.277–0.740)	0.002		
HCV therapy	Yes vs. No					0.239 (0.129–0.445)	< 0.001

*NSAIDs* Nonsteroidal anti-inflammatory drugs, *NA therapy* Nucleos(t)ide analogues

HCV therapy included interferon and direct-acting antiviral medications

In subgroup analysis, DM, Child-Pugh grade and vascular invasion were significantly associated with poor OS among CHB patients. Whereas, NA therapy was related to better OS. Among CHC patients, liver cirrhosis, diabetes and vascular invasion were related to poor OS, while HCV therapy was associated with better OS.

### Patient RFS and OS evaluation using propensity score-matching analysis

**Fig. 5:**
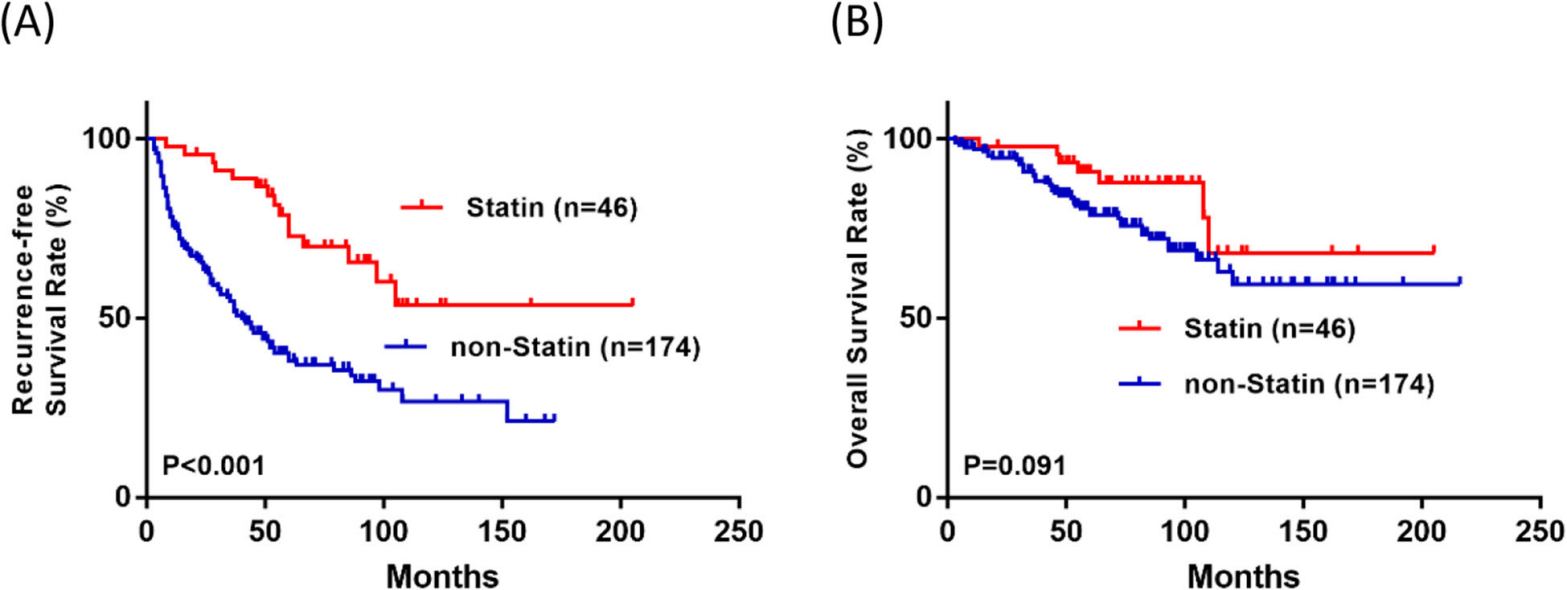
Kaplan-Meier cumulative recurrence-free survival **a** and overall survival **b** curves for propensity score-matched patients stratified by statin use

After 1:4 case propensity score matching, 46 patients in the stain group and 174 patients in the non-statin group were analyzed. The baseline characteristics were balanced between the matched groups (SMD < 0.2 and *p* > 0.05 for all variables). The patient characteristics before and after matching are presented in Table 4. The RFS rate was significantly higher in the statin than non-statin group (*p* < 0.001, Fig. 5a). Moreover, statin use remained significantly associated with a reduced risk of HCC recurrence after PSM (HR: 0.328; CI: 0.190–0.566; *p* < 0.001; Table 5). The OS rate was not significantly different between the groups after PSM (Fig. 5b and Table 6).

**Table 4:** Patient Characteristics in the Propensity Model

Variable	Statin (*n =* 46)	Non-statin (*n =* 174)	*P*-value	SMD
Age, years; mean (SD)	62.02 (8.14)	62.38 (9.35)	0.957	0.041
Sex			0.975	0.005
Male	40 (87.0%)	151 (86.8%)		
Female	6 (13.0%)	23 (13.2%)		
Diabetes	27 (58.7%)	94 (54.0%)	0.572	0.094
HBV	22 (47.8%)	78 (44.8%)	0.717	0.059
HCV	14 (30.4%)	56 (32.2%)	0.821	0.037
Liver cirrhosis	12 (26.1%)	51 (29.3%)	0.668	0.072
Child-Pugh grade			0.826	0.037
A	42 (91.3%)	157 (90.2%)		
B	4 (8.7%)	17 (9.8%)		
Tumor size (> 2 cm)	42 (91.3%)	162 (93.1%)	0.677	0.067
Tumor number			0.449	0.114
Single	44 (95.7%)	170 (97.7%)		
Multiple	2 (4.3%)	4 (2.3%)		
Vascular invasion	22 (47.8%)	77 (44.3%)		0.071

*SMD* Standardized mean difference, *HBV* Hepatitis B virus, *HCV* Hepatitis C virus

**Table 5:** Univariate and multivariate analysis for recurrence after curative hepatectomy for propensity score-matched patients with BCLC 0/A stage HCC

Variable	Comparison	Univariate		Multivariate	
		HR (95% CI)	*P* value	HR (95% CI)	*P*-value
Age (years)	>60 vs. ≦ 60	1.013 (0.696–1.473)	0.947		
Sex	Male vs. Female	0.933 (0.557–1.561)	0.791		
AFP (ng/mL)	>200 vs. ≦ 200	0.785 (0.456–1.351)	0.381		
Liver cirrhosis	Yes vs. No	1.436 (0.982–2.102)	0.062		
Diabetes	Yes vs. No	1.733 (1.194–2.517)	0.004	1.807 (1.241–2.632)	0.002
Child-Pugh grade	B vs. A	1.345 (0.756–2.395)	0.313		
Tumor number	Multiple vs. Single	1.374 (0.506–3.729)	0.533		
Tumor size (cm)	>2 vs. ≦ 2	1.854 (0.819–4.243)	0.138		
Histology stages	poor vs. well + moderate	1.143 (0.652–2.004)	0.640		
Vascular invasion	Yes vs. No	1.511 (1.046–2.184)	0.028	1.446 (0.999–2.093)	0.051
Statin	Yes vs. No	0.328 (0.190–0.566)	< 0.001	0.304 (0.176–0.525)	< 0.001
NSAID	Yes vs. No	1.379 (0.606–3.318)	0.443		
Aspirin	Yes vs. No	0.689 (0.401–1.184)	0.178		
Metformin	Yes vs. No	0.811 (0.508–1.296)	0.381		
NA therapy	Yes vs. No	0.670 (0.424–1.057)	0.085		
HCV therapy	Yes vs. No	0.963 (0.568–1.634)	0.890

**Table 6:** Univariate and multivariate analysis for overall survival after curative hepatectomy for propensity score-matched patients with BCLC 0/A stage HCC

Variable	Comparison	Univariate		Multivariate	
		HR (95% CI)	*P-*value	HR (95% CI)	*P-*value
Age (years)	>60 vs. ≦ 60	0.990 (0.558–1.757)	0.974		
Sex	Male vs. Female	1.013 (0.455–2.253)	0.975		
AFP (ng/mL)	>200 vs. ≦ 200	1.172 (0.545–2.518)	0.685		
Liver cirrhosis	Yes vs. No	1.669 (0.942–2.955)	0.079		
Diabetes	Yes vs. No	3.175 (1.652–6.103)	0.001	2.942 (1.514–5.717)	0.001
Child-Pugh grade	B vs. A	1.415 (0.601–3.330)	0.427		
Tumor number	Multiple vs. Single	0.711 (0.098–5.159)	0.736		
Tumor size (cm)	>2 vs. ≦ 2	4.271 (0.588–30.998)	0.151		
Histology stages	poor vs. well + moderate	1.651 (0.651–4.187)	0.291		
Vascular invasion	Yes vs. No	2.455 (1.367–4.409)	0.003	1.979 (1.092–3.588)	0.025
Statin	Yes vs. No	0.509 (0.229–1.132)	0.098		
NSAID	Yes vs. No	3.305 (1.309–8.345)	0.011	3.343 (1.303–8.580)	0.012
Aspirin	Yes vs. No	1.021 (0.479–2.176)	0.957		
Metformin	Yes vs. No	1.310 (0.669–2.566)	0.431		
NA therapy	Yes vs. No	0.830 (0.425–1.622)	0.586		
HCV therapy	Yes vs. No	0.440 (0.137–1.418)	0.169		

## Discussion

Liver resection remains the mainstay of curative treatment for early-stage HCC with preserved liver function; however, the 5-year cumulative recurrence rates after resection are higher than 50% [9]. Certain medications, including statins, aspirin, NSAIDs and metformin, have been reported to alter the risk of developing HCC [14, 17, 26–28]. However, the effects of these medications on HCC recurrence have not yet been examined. In this population-based, propensity score-matched study, we confirmed that statin use may lower the risk of HCC recurrence in patients with HCC after curative resection. This association remained consistent regardless of age, sex, cause of hepatitis, diabetic status or the presence or absence of cirrhosis, which suggests statins could be beneficially employed as a chemopreventive agent to reduce the risk of recurrence after resection in patients with HCC. These results emphasize the need for large-scale RCTs to validate the potential chemopreventive effect of statins on the recurrence of HCC.

Statins, 3-hydroxy-3-methylglutaryl coenzyme A (HMG-CoA) reductase inhibitors, are used worldwide as a treatment for dyslipidemia and can prevent cardiovascular events and mortality [16, 29]. In addition to their cholesterol-lowering capability, increasing evidence indicates statins also exert anti-oncogenic effects. Kim et al. reported that statin use decreased the risk of developing HCC among patients with new-onset type 2 diabetes mellitus in a nested case-control, longitudinal study [30]. Tsan et al. demonstrated that statins may dose-dependently reduce the risk of HCC among individuals with HBV or HCV infection [17, 19]. Furthermore, a recent meta-analysis of 25 studies that included 1,925,964 patients concluded statins exert a beneficial chemopreventive effect against the development of HCC [31].

However, most of these studies focused on the ability of statins to protect against the development of HCC; only a few studies have assessed the potential of statins to protect against recurrence after curative resection. A retrospective study in Japan by Kawaguchi et al. showed that statins may protect against HCC recurrence [32]. Similarly, we found statin use was associated with a significantly lower risk of recurrence after resection (HR: 0.34; *p* = 0.005). However, OS, including liver- and non-liver-related mortality, were not significantly different between the statin and non-statin groups in this study (Supplementary Figure 2). The differences between the study by Kawaguchi et al. and our findings may be related to the varied proportions of patients with HBV and HCV infection. In the study by Kawaguchi et al., significantly fewer patients in the statin group had hepatitis B surface antigen (HBsAg) positivity and hepatitis C virus antibody (HCVAb) positivity compared to the non-statin group (HBsAg: 6.5% vs. 22.8%, *p* = 0.032; HCVAb: 19.4% vs. 45.0%, *p* = 0.005). In the present study, there was no difference in the proportions of HBV- and HCV-positive patients between the statin and non-statin groups. More importantly, we also compared various potential chemopreventive agents, including statins, aspirin, metformin and NSAIDs. To the best of our knowledge, this study represents the largest analysis of the relationship between chemopreventive agents and HCC recurrence in a country where HBV and HCV are endemic.

The mechanisms underlying the ability of statins to protect against HCC development are not well understood; some potential mechanisms have been suggested. First, statin-mediated reduction of downstream metabolites of the mevalonate pathway—including geranyl pyrophosphate, farnesyl pyrophosphate and geranylgeranyl pyrophosphate—interferes with cancer cell proliferation and differentiation, which promotes apoptosis [33, 34]. Secondly, statins can suppress proteasomal degradation, which limits breakdown of the cyclin-dependent kinase (CDK) inhibitors p21 and p27 and reduces CDK2 expression, and thus disrupts mitosis in malignant cells [35, 36]. Third, statins may inhibit tumor cell migration and invasion by attenuating angiogenesis via downregulating VEGF production [37]. Fourth, statins exert anti-inflammatory and immunomodulatory effects by decreasing TNF-α and IL-6 expression, downregulating the activity of metalloproteinases, and inducing a shift towards the TH2 cytokine anti-inflammatory response, which may reduce hepatic inflammation [38, 39]. Chronic hepatic inflammation plays an important role in hepatocarcinogenesis [40]. Moreover, statins activate AMP-activated protein kinase, which enhances p21 expression and the endoplasmic reticulum stress response, and thus induces higher levels of autophagy [41].

Statins are generally classified into hydrophilic and lipophilic groups based on tissue selectivity. Lipophilic statins, including atorvastatin, simvastatin, lovastatin, fluvastatin and pitavastatin, distribute widely throughout various tissues. Hydrophilic statins, such as pravastatin and rosuvastatin, have lower levels of tissue absorption—except in the liver—and exert fewer side effects as they are not metabolized by cytochrome P450 enzymes [42]. Although a previous meta-analysis showed lipophilic statins, but not hydrophilic statins, were associated with a lower risk of developing HCC, we did not observe a significant difference in RFS between the subgroups of patients taking lipophilic and hydrophilic statins (Supplementary Figure 1). However, this analysis may be affected by the limited number of patients. Furthermore, the mechanisms that explain the varied anticancer efficacies of lipophilic and hydrophilic statins remain to be determined.

A recent cohort study by Young et al. indicated aspirin use—but, interestingly, not statin use—reduced the risk of HCC recurrence [43]. In contrast, aspirin use was not significantly associated with HCC recurrence in our cohort (*p* = 0.864). These discrepancies may be related to differences between the design of each study. Firstly, Young et al. only examined exposure to chemopreventive agents in the 30 days before tumor recurrence. However, we defined exposure as more than 90 days, as generally adopted by previous studies [32, 44]. Secondly, Young et al. enrolled patients with BCLC stage A/B/C HCC who underwent resection. In contrast, we only assessed patients with BCLC stage 0/A, so called early-stage HCC, for which surgical resection is the widely accepted standard treatment. Moreover, Young et al. focused on HBV-related HCC, while we investigated all etiologies. Since no RCTs have been published in this field, our results further emphasize the need for large-scale RCTs to validate the potential chemopreventive effect of statins on HCC recurrence.

We found that age, liver cirrhosis, diabetes, number of tumors, tumor size and vascular invasion represented the major risk factors for HCC recurrence, and antiviral therapy may reduce the risk of HCC recurrence. These results are consistent with previous reports [7, 9, 11–14, 45].

Increasing evidence indicates that gut microbiota alterations promote the development of HCC by inducing a leaky gut and gut dysbiosis; both of which are prominent features of all stages of chronic liver disease, and promote the stepwise progression from fibrosis to cirrhosis and HCC [46]. In addition to dysbiosis, gut microbiota-derived metabolites may also promote hepatocarcinogenesis via a variety of metabolic pathways [47]. Although there is no evidence to prove statin use affects HCC development and recurrence by altering the human gut microbiome, several studies have indicated statin therapy lowers the prevalence of gut microbiota dysbiosis [48] and also affects the virulence and growth of bacterial pathogens in microbial infections [49, 50]. Therefore, we hypothesize that statin use may affect the human gut microbiome, and in turn directly or indirectly reduce hepatocarcinogenesis via the gut-liver axis. Further animal experiments are required to delineate the effects of statins on the development and recurrence of HCC through the gut-microbiota-liver axis.

There are some limitations to this study. First, this was a retrospective study of patients from a single institution and the data were collected from medical records. Despite the use of multivariable analysis and propensity score-matching analysis, not all confounding factors can be completely adjusted for. Secondly, the number of patients was relatively low. There were 46 (5.6%) patients in the statin group; however, this is comparable to the study in Japan (31/734, 4.2%) and may reflect the real-world situation. Finally, we could not obtain information on tobacco use and alcohol consumption, which may also be risk factors in survival analysis. Ultimately, a large randomized trial of a suitable regimen in well-selected patients treated using standard approaches is required to obtain this important information.

## Conclusions

In summary, statin use may exert a chemopreventive effect on HCC recurrence after curative resection. Further prospective randomized controlled studies are needed to confirm these observations.

## Supplementary Information

**Additional file 1: Supplementary Figure 1.** Kaplan-Meier cumulative recurrence-free survival curves for patients with HCC using statins stratified by (A) lipophilic or hydrophilic statins and (B) individual statins.

**Additional file 2: Supplementary Figure 2.** Kaplan-Meier (A) liver-related survival and (B) non-liver-related survival curves after curative resection for patients with HCC stratified by statin use.

## Data Availability

The original data are available upon reasonable request to the corresponding author.

## Abbreviations

HCC: Hepatocellular carcinoma
BCLC: Barcelona Clinic Liver Cancer
OS: Overall survival
RFS: Recurrence-free survival
HR: Hazard ratio
HBV: Hepatitis B virus
HCV: Hepatitis C virus
RFA: Radiofrequency ablation
NA: Nucleos(t)ide analogue
HMG-CoA: 3-hydroxy-3-methyglutaryl-coenzyme A
DDD: Defined daily dose
AFP: Alpha-fetoprotein
HBsAg: Hepatitis B surface antigen
HCVAb: Hepatitis C virus antibody
CDK: Cyclin-dependent kinase
SMD: Standardized mean difference
NSAIDs: Nonsteroidal anti-inflammatory drugs
