# Supplementary material for: Statin use is associated with a lower risk of recurrence after curative resection in BCLC stage 0-A hepatocellular carcinoma
Source: BMC Cancer. 2021 Jan 15;21:70. doi: 10.1186/s12885-021-07796-7 (PMC7808883; doi:10.1186/s12885-021-07796-7)

Supplementary Figure 1. Kaplan-Meier cumulative recurrence-free survival curves for patients with HCC using statins stratified by (A) lipophilic or hydrophilic statins and (B) individual statins.

(A)


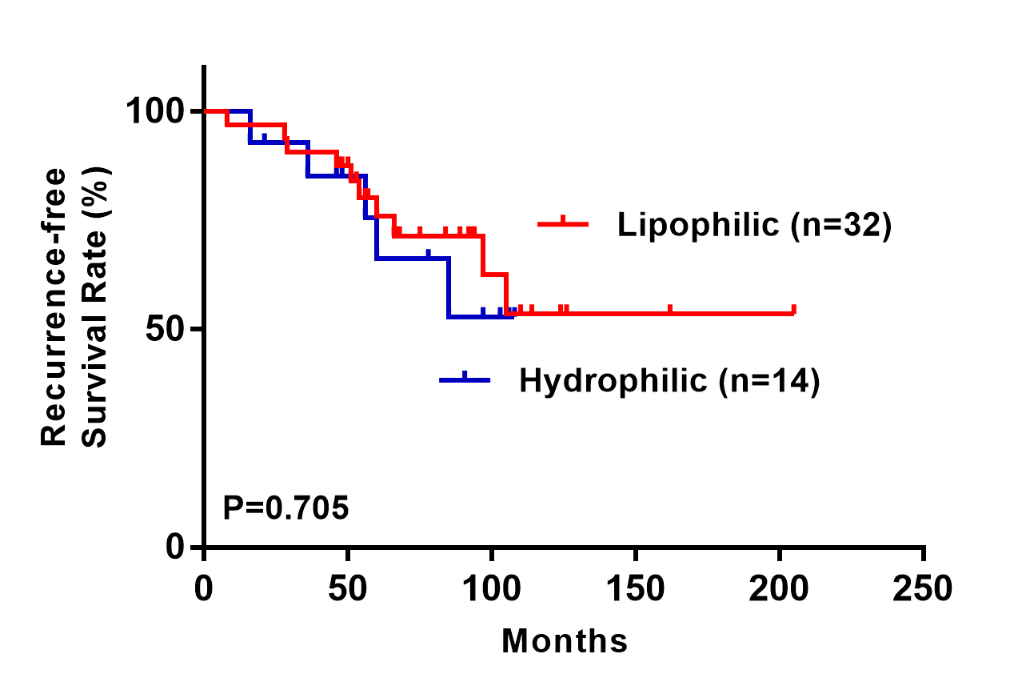


(B)


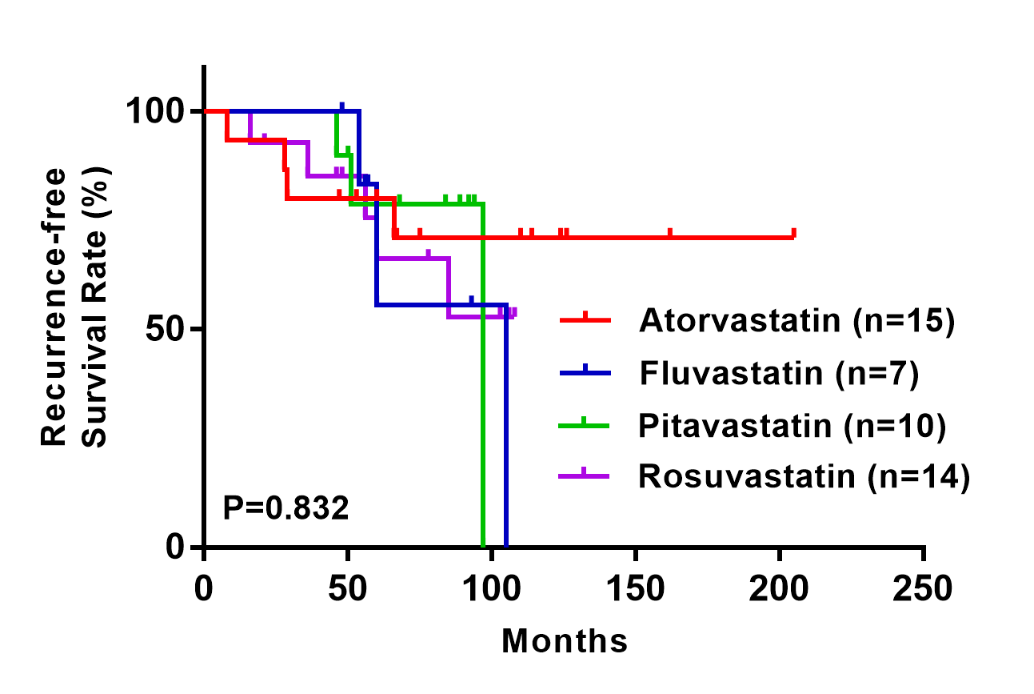


Supplementary Figure 2. Kaplan-Meier (A) liver-related survival and (B) non-liver-related survival curves after curative resection for patients with HCC stratified by statin use.

(A)


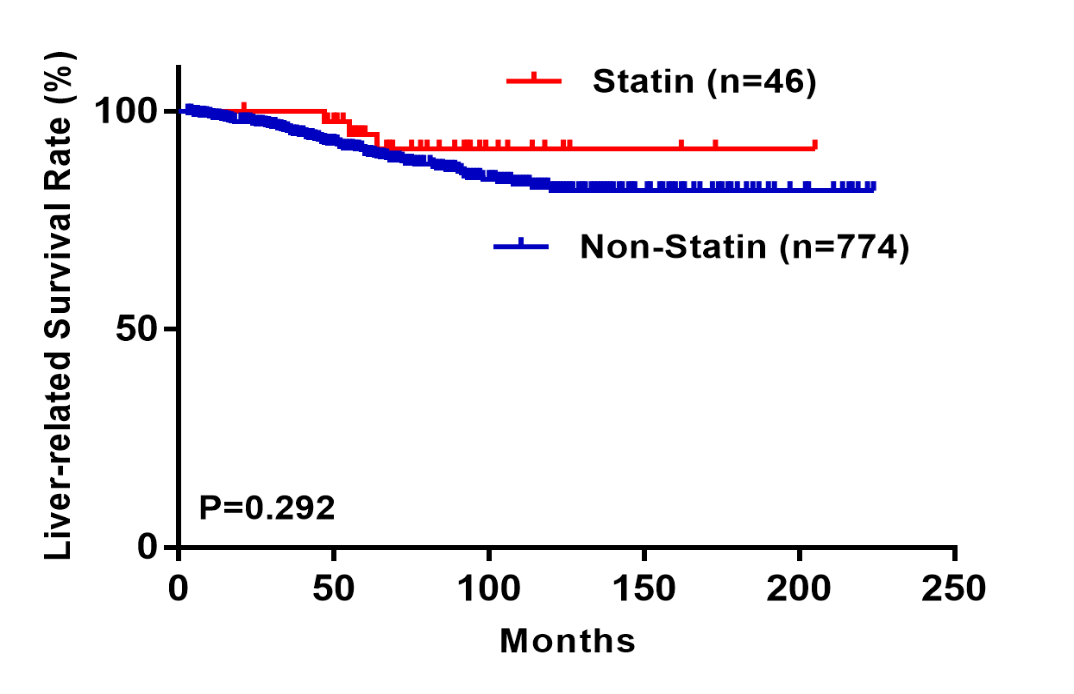


(B)


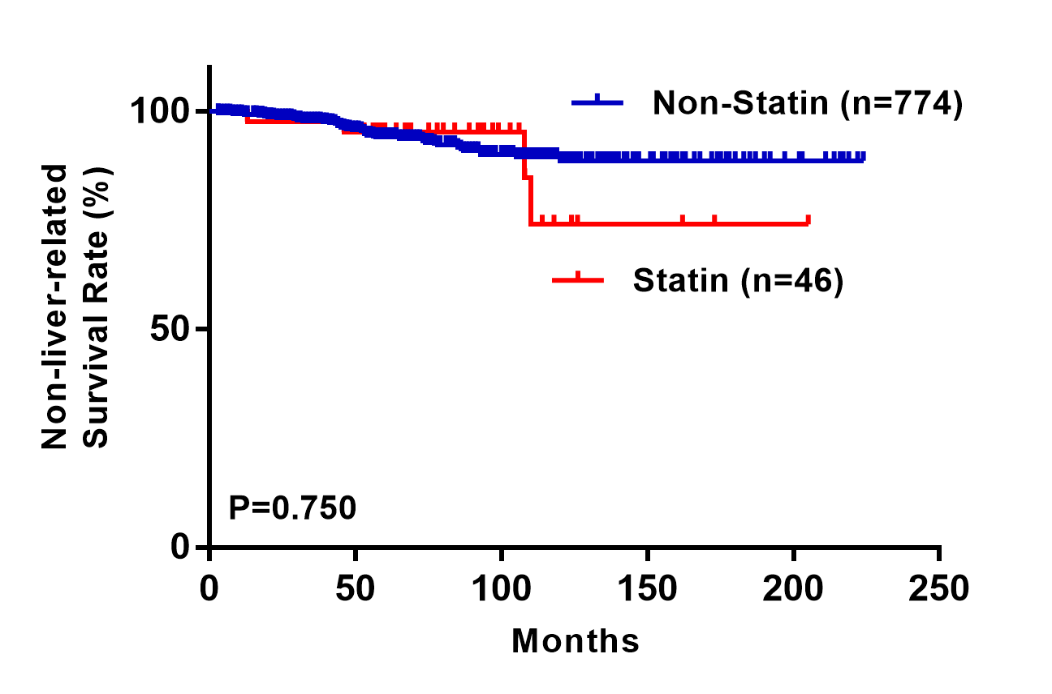

Supplement: Supplementary file 1 — Additional file 1: Supplementary Figure 1. Kaplan-Meier cumulative recurrence-free survival curves for patients with HCC using statins stratified by (A) lipophilic or hydrophilic statins and (B) individual statins. [file 12885_2021_7796_MOESM1_ESM.docx]
